# Platelet-activating factor mediates trinitrobenzene induced dysmotility in the left colon

**DOI:** 10.1155/S0962935195000032

**Published:** 1995-01

**Authors:** W. E. Longo, J. Standeven, B. Chandel, Y. Deshpande, A. M. Vernava, D. Kaminski

**Affiliations:** 1 Department of Surgery The Surgical Research Institute St Louis University School of Medicine St Louis MO USA

## Abstract

We investigated whether TNB alters colonic tissue levels of PAF at the expense of changes in colonic motility in the isolated perfused rabbit left colon. Colonic inflammation was induced by the intracolonic administration of 0.25 ml of 50% ethanol containing 30 mg of trinitrobenzene. Strain gauge transducers were sewn onto the serosal surface of the colon to evaluate colonic motility. TNB administration increased tissue levels of PAF and increased the average force of each colonic contraction. Pretreatment with the PAF receptor antagonist WEB-2170 prior to TNB infusion decreased tissue concentrations of PAF and ameliorated the altered motility parameters seen with TNB alone. These results suggest that PAF is stimulated by TNB and may participate in colonic dysmotility during inflammatory states.


Short Communication

Mediators of Inflammation 4, 17–18 (1995)

### Platelet-activating factor mediates trinitrobenzene induced dysmotility in the left colon

W. E. Longo,[CA] J. Standeven, B. Chandel, Y. Deshpande, A. M. Vernava and D. Kaminski

Department of Surgery, The Surgical Research Institute, St Louis University School of Medicine, St Louis, MO, USA

[CA] Corresponding Author

**We investigated whether TNB alters colonic tissue levels of PAF at the expense of changes in colonic motility in the isolated perfused rabbit left colon. Colonic inflammation was induced by the intracolonic administration of 0.25 ml of 50% ethanol containing 30 mg of trinitrobenzene. Strain gauge transducers were sewn onto the serosal surface of the colon to evaluate colonic motility. TNB administration increased tissue levels of PAF and increased the average force of each colonic contraction. Pretreatment with the PAF receptor antagonist WEB-2170 prior to TNB infusion decreased tissue concentrations of PAF and ameliorated the altered motility parameters seen with TNB alone. These results suggest that PAF is stimulated by TNB and may participate in colonic dysmotility during inflammatory states.**

**Key words**: Motility, Platelet-activating factor.

Several mediators are involved in the wide spectrum of disease states that affect the large bowel both in models of experimental colitis and in *in vivo* profiles of tissue obtained from patients with inflammatory bowel disease and other forms of colitis. The colonic generation of eicosanoids, autacoids such as platelet-activating factor as well as cytokines have been implicated in the inflammatory response.[1] Models of experimental colitis have not only produced acute inflammation, but also demonstrated disturbances in colonic motility.[2,3] Results from this laboratory have demonstrated that exogenous PAF produces not only colonic inflammation similar to that seen in inflammatory bowel disease, but induces dysmotility in the colon mediated by neuropeptides.[4] The aim of the present study was to evaluate the possible role of endogenous PAF in the dysmotility changes of the left colon of the rabbit to trinitrobenzene sulphonic acid (TNB), a hapten which produces acute mucosal inflammation and chronic transmural inflammation in experimental colitis.

The experimental procedure for producing colonic inflammation by PAF on the isolated rabbit colon has been reported previously.[5] After isolating the colon, it was placed on a temperature controlled base, humidified whole organ perfusion apparatus (Mx International, Aurora, CO) and allowed to equilibrate with an intra-arterial Krebs–Ringer's bicarbonate (KRB) infusion for the first 30 min period. This was followed by the intraluminal infusion of 0.25 ml of 50% ethanol containing 30 mg of TNB, while continuing infra-arterial infusion of KRB for 30 min. At the end of this period, TNB was discontinued and the colon was allowed to recover for 30 min, infusing only KRB intra-arterially. Separate studies were performed in which the colons were pretreated intra-arterially with $10^{-9}$ M of the PAF antagonist WEB-2170 30 min prior to administration of TNB. Strain gauge transducers were sewn onto the serosal surface of the colon to measure colonic motility. Motility was calculated as four indices: (1) contractions/min; (2) peak force per contraction measured in grams; (3) average force per contraction; and (4) the minute motility index (MMI) calculated as the sum of the contractions, weighed by the peak force, expressed as a per minute average. At the end of the experiment, tissue samples of colon were taken and immersed in liquid nitrogen and then stored at –70°C. Tissue levels of PAF were determined by a well-described radioimmunoassay.[6]

Tissue concentrations of PAF in TNB treated colons were significantly increased compared with control values. When administered prior to TNB, WEB-2170 significantly decreased the tissue concentrations of PAF. Motility results demonstrated that TNB did not affect contractility, decreased the peak force and increased the average force per contraction. Similarly there was a trend toward decreasing the MMI. Pretreatment of colons with WEB-2170 prior to TNB administration resulted in peak force, average force and MMI values to return to that seen with KRB infusion alone (Table 1).

Colonic inflammation has a complex aetiology inter-related with the immune system, the neuroendocrine system and aracidonic acid metabolism. The interaction of these phenomena with changes in colonic motility has attracted our attention recently. Endogenous release of PAF is felt to be partly responsible for the intestinal motor alterations induced by endotoxin which are strongly reduced after treatment with a PAF antagonist, and suggested to be mediated through the release of prostaglandins.[7] Two recent study of colitis induced

© 1995 Rapid Communications of Oxford Ltd

**Table 1.** The effect of TNB on tissue PAF and colonic motility

| | KRB ($n = 5$) | NB ($n = 5$) | WEB-2170 + TNB ($n = 5$) |
|---|---|---|---|
| Tissue PAF levels[a] | 95.5 ± 12.7 | 294.8 ± 19.6* | 124.8 ± 31.3** |
| Contractions/min | 23.5 ± 1.9 | 25.3 ± 3.7 | 22.1 ± 4.1 |
| Peak force[b] | 0.77 ± 0.07 | 0.45 ± 0.06* | 0.83 ± 0.06** |
| Average force[c] | 0.44 ± 0.3 | 0.62 ± 0.08* | 0.36 ± 0.05** |
| MMI[d] | 37.4 ± 5.5 | 29.3 ± 6.3 | 35.7 ± 4.6 |

* $p < 0.05$, TNB and KRB; **$p < 0.05$, WEB-2170 + TNB and TNB. [a] pg/mg wet tissue. [b] peak force per contraction. [c] Average force per contraction. [d] Minute Motility Index.

by TNB have been described in *in vivo* rat models of colitis and motility alterations. TNB instilled in the proximal colon of rats induced immediate disturbances of colonic motility which were abolished by the administration of a leukotriene antagonist.[2] In a similar study the effects of TNB on the proximal colon were investigated using cyclooxygenase and 5-lipoxygenase pathway inhibitors, cytokine antagonists and PAF antagonists. It was demonstrated that TNB-induced colitis in rats involves dysmotility involving PAF and IL-1, but not other eicosanoids.[3] Our data suggest that PAF is stimulated by TNB and may participate in colonic dysmotility disturbances during inflammatory states of the left colon. Studies in an effort to determine whether endogenously stimulated PAF by TNB results in neuropeptide release similar to that seen with intra-arterial PAF infusion[4] are currently in progress.

### References

1. Lauritsen K, Laursen LS, Bukhave K, Rask-Madsen J. *In vivo* profiles of eicosanoids in ulcerative colitis, Crohn's colitis and *Clostridium difficile* colitis. *Gastroenterology* 1988; **95**: 11–17.
2. Pons L, Droy-Lefaix MT, Bveno L. Leukotriene D4 participates in colonic transit disturbances induced by intracolonic administration of trinitrobenzene sulfonic acid in rats. *Gastroenterology* 1992; **102**: 149–156.
3. Morteav O, More J, Pons L, Lveno L. Platelet-activating factor and interleukin-1 are involved in colonic dysmotility in experimental colitis in rats. *Gastroenterology* 1993; **104**: 47–56.
4. Deshpande Y, Longo WE, Chandel B, *et al.* Effect of platelet-activating factor (PAF) and its antagonists on colonic dysmotility and tissue levels of colonic neuropeptides. *Eur J Pharmacol* 1994; **256**: 121–123.
5. Chandel B, Niehoff M, Deshpande Y, Standeven J, Vernava AM, Kaminski DL, Longo WE. The isolated perfused colon: a novel model to study colonic inflammation and motility. *Surg Research Comm* (in press).
6. Kaminski DL, Andrus CH, German D, Deshpande YG. The role of prostanoids in the production of acute acalculous cholecystitis by platelet-activating factor (PAF). *Ann Surg* 1990; **212**: 455–461.
7. Pons L, Droy-Lefaix MT, Braquet P, Bueno L. Involvement of platelet-activating factor (PAF) in endotoxin-induced intestinal motor disturbances in rats. *Life Sciences* 1989; **45**: 533–541.

**Received 9 September 1994; accepted 15 September 1994**
